# Adoptive Transfer of Ceramide Synthase 6 Deficient Splenocytes Reduces the Development of Colitis

**DOI:** 10.1038/s41598-017-15791-x

**Published:** 2017-11-14

**Authors:** Matthew J. Scheffel, Kristi Helke, Ping Lu, Jacob S. Bowers, Besim Ogretmen, Elizabeth Garrett-Mayer, Chrystal M. Paulos, Christina Voelkel-Johnson

**Affiliations:** 1 Department of Microbiology & Immunology, Medical University of South Carolina, Charleston, SC USA; 2 Department of Comparative Medicine, Medical University of South Carolina, Charleston, SC USA; 3 Department of Biochemistry & Molecular Biology, Medical University of South Carolina, Charleston, SC USA; 4 Department of Public Health Sciences, Medical University of South Carolina, Charleston, SC USA

## Abstract

Sphingolipids regulate critical cellular processes including inflammation. Ceramide, which serves a central role in sphingolipid metabolism, is generated by six ceramide synthases (CerS) that differ in substrate specificity. CerS6 preferentially generates C_16_-ceramide and its mRNA is highly expressed in immune tissues. In this study we analyzed how deficiency of CerS6 impacts on the development of colitis using an adoptive transfer model. Adoptive transfer of CerS6-deficient splenocytes, which have significantly decreased levels of C_16_-ceramide, showed that CerS6-deficiency protected against the development of colitis. However, adoptively transferred cells isolated from the lamina propria of the large intestine from wild type or CerS6-deficient groups showed no differences in the percentages of immune-suppressive regulatory T cells, pro-inflammatory Th17 cells, or their ability to express IL-17. *In vitro* polarization of wild type or CerS6-deficient splenocytes also revealed no defects in the development of T cell subsets. Our data suggest that protection from colitis following adoptive transfer of CerS6-deficient splenocytes maybe related to their ability to migrate and proliferate *in vivo* rather than subset development or cytokine expression.

## Introduction

Sphingolipids are a lipid subtype that can regulate critical processes such as cell growth and death, autophagy, migration and invasion, angiogenesis and inflammation^1,2^. Central to sphingolipid metabolism is ceramide, which varies in acyl chain length ranging from 14–30 (or more) carbons^3^. This variety in acyl chain length derives from the activity of six ceramide synthases (CerS) that have different substrate preferences^4^. Through the combined activity of multiple CerS, cells are endowed with a specific ceramide profile that likely reflects the need for proper signaling and sphingolipid homeostasis in any given tissue. Changes in ceramide profiles have been observed in irritable bowel syndrome, Alzheimer’s disease, cystic fibrosis, multiple sclerosis, and cancer, suggesting that dysregulation of CerS may contribute to onset or progression in a variety of diseases^5,6^.

The development of CerS-deficient mice has shed some light onto the roles and functions of specific ceramides and suggests that ceramide fatty acid length is associated with distinct biological roles. For example, CerS3 or CerS4-deficient mice develop problems with skin barrier function and alopecia, respectively^7,8^, while CerS1- and CerS2-deficient mice exhibit abnormalities in the central nervous system^5^. CerS2-deficiency (inability to generate C_24_-Cer) results in a compensatory generation of C_16_-Cer, which in the liver leads to apoptosis and subsequent development of hepatocellular cancer^9,10^. Lack of CerS5/6 expression impacts metabolism in a diet-induced obesity model and deficiency of CerS6 can also result in behavioral defects^11–13^. Altered expression of CerS2, CerS5, or CerS6 can impact cytokine production and inflammation^12,14–17^. As CerS6 mRNA is strongly expressed in cells of the immune system, we investigated how CerS6-deficiency impacts on the development of colitis in an adoptive transfer model^4^.

## Results

### Characterization of CerS6-deficient mice

**Figure 1:**
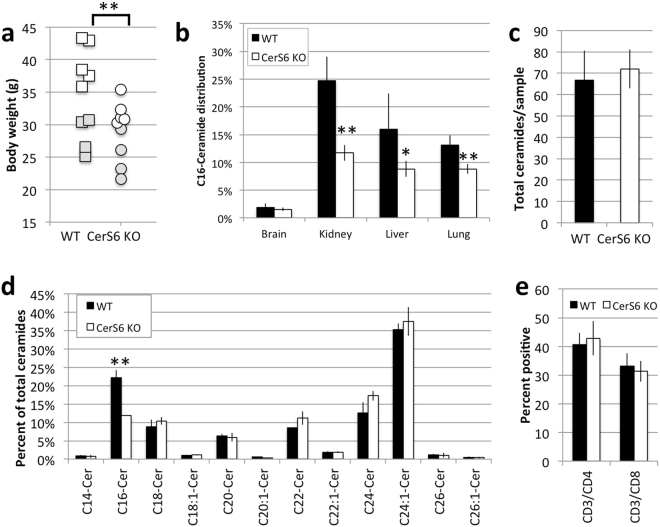
Characterization of CerS6-deficient mice. (**a**) Comparison in body weight among 9 pairs of wild type and CerS6 gender matched littermates (4 pairs of females, 5 pairs of males) ranging in age from 167 to 315 days. Males and females are indicated by white and grey symbols, respectively. (**b**) Distribution of ceramides in wild type and CerS6 deficient mice (n = 4–5). (**c**,**d**) Total ceramide and distribution of ceramides among splenocytes (n = 2). Similar results observed in 3 experiments. (**e**) Analysis of CD3^+^CD4^+^ and CD3^+^CD8^+^ cells among wild type and CerS6-deficient splenocytes, n = 7. *p < 0.05; **p < 0.005.

CerS6-deficient mice were generated on a C57BL6 background by the Texas Institute for Genomic Medicine. In our experience homozygous breeding of CerS6-deficient mice is difficult as pups often fail to thrive. Therefore all experiments were performed with wild type and CerS6-deficient littermates derived from heterozygous parents. CerS6-deficient mice tended to be significantly smaller in overall body size than their wild type littermates (Fig. 1a). However, when separated by gender the difference was only significant in males (p < 0.05, n = 5). Analysis of ceramides from lung, liver, and kidney revealed a significant decrease in C_16_-ceramide, which in liver and kidney was accompanied by an increase in C_24:1_-ceramide (Fig. 1b, Fig. S1, Table S1). Splenocytes did not differ in total ceramide content but CerS6-deficiency resulted in a significant decrease in C_16_-ceramide compared to wild type cells (Fig. 1c,d). This decrease in C_16_-ceramide did not affect the proportion of splenic CD4^+^ and CD8^+^ T cells (Fig. 1e) or memory phenotype (Fig. S2). Levels of sphingosine-1-phosphate (S1P), which can functionally oppose ceramide, were comparable (2.5 ± 0.2 vs. 2.6 ± 0.1 pmol/20 million cells in wild type and CerS6-deficient splenocytes, respectively).

### Adoptive transfer of CerS6-deficient naïve CD4 cells protects against colitis

**Figure 2:**
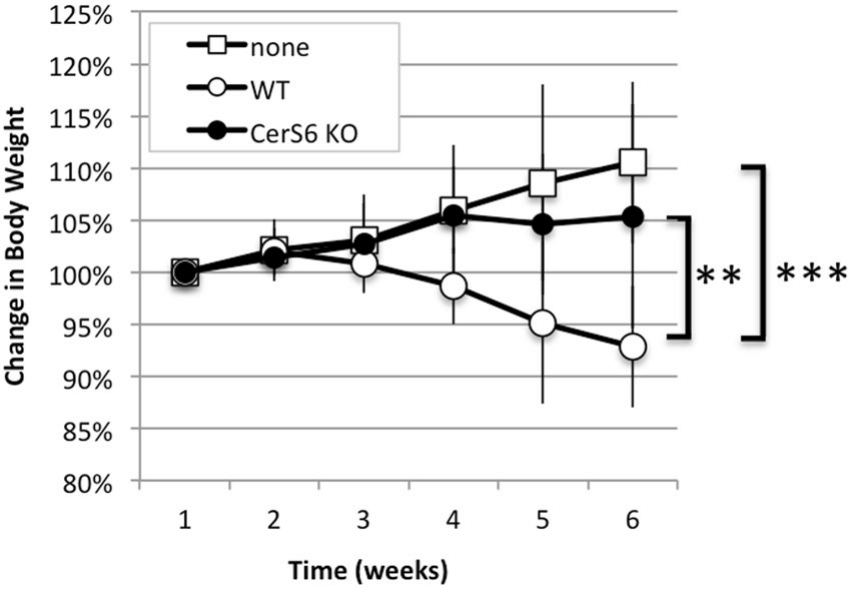
Body weight following adoptive transfer of naïve CD4^+^ T cells. Body weight was monitored weekly in mice that received no transfer of cells (none, n = 3), wild type cells (WT, n = 6), or CerS6-deficient cells (KO, n = 6). Statistical differences after week 3 were p < 0.0001 (**, none vs. WT) and p < 0.005 (*, WT vs. KO). None and KO were not significantly different.

To determine whether CerS6 splenocytes differed in their ability to induce colitis, we isolated naïve CD4^+^CD45RB^hi^ cells from either wild type or CerS6-deficient mice and adoptively transferred them into RAG1-deficient recipients. Three weeks after adoptive transfer mice receiving wild type but not CerS6-deficient cells experienced significant weight loss that continued until the end of the experiment (Fig. 2).

**Figure 3:**
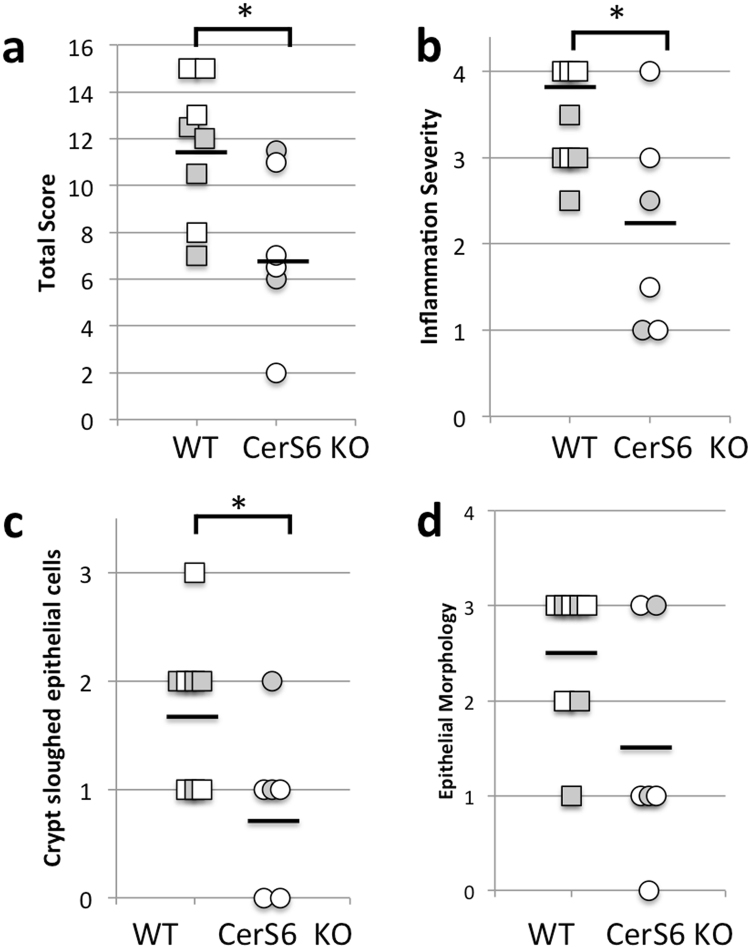
Histological analysis of the large intestine. (**a**) Total inflammatory scores, (**b**) inflammation severity, (**c**) crypt sloughed epithelial cells and (**d**) epithelial morphology following adoptive transfer of wild type or CerS6-deficient naïve CD4^+^ splenocytes. *p < 0.05, n = 6–8. Males and females are indicated by white and grey symbols, respectively.

**Figure 4:**
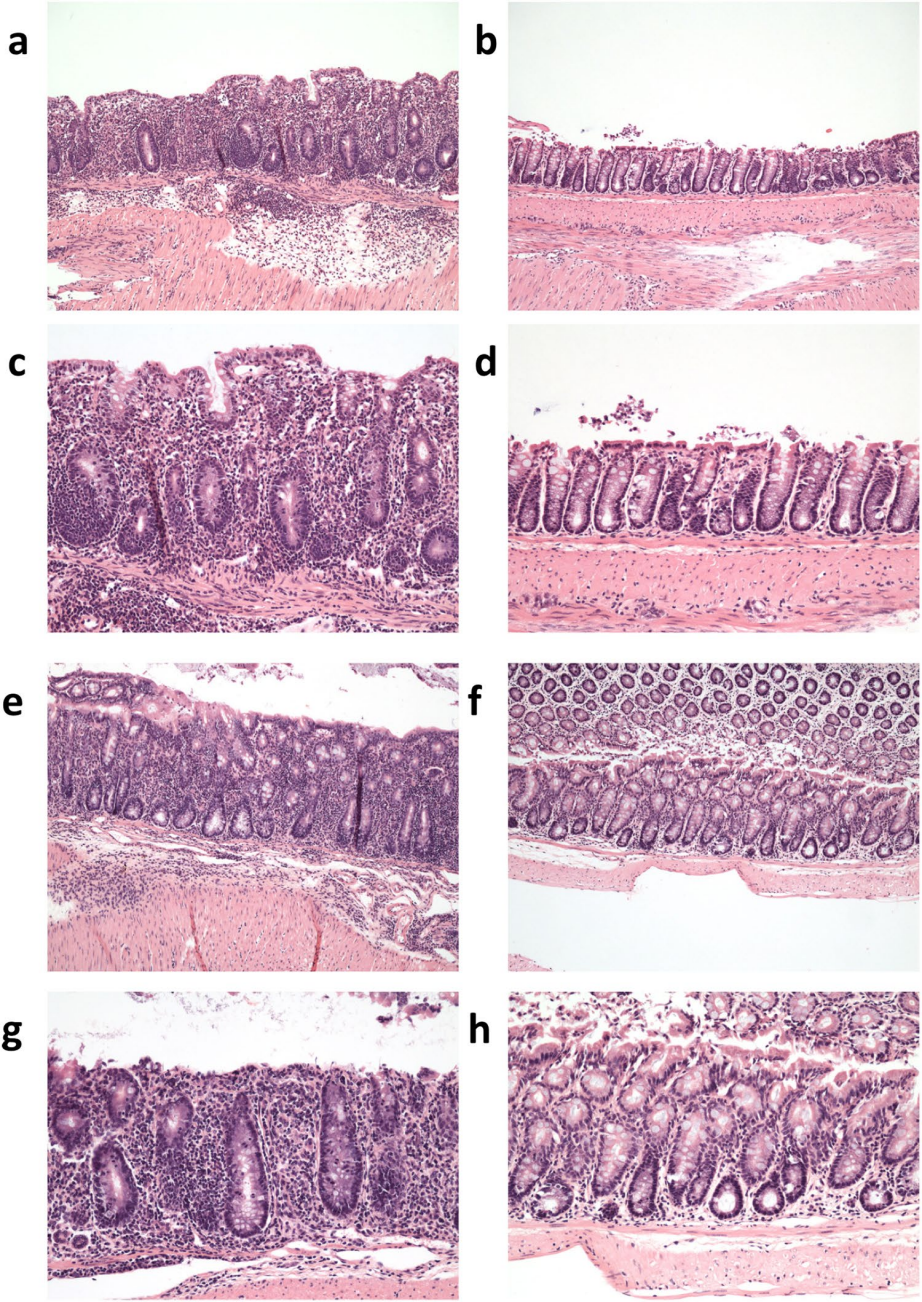
Histopathology in of the large intestine following transfer of wild type and CerS6-deficient naïve CD4^+^ T cells. Panels a–d show histology of males that received adoptive transfer of wild type (**a,c**) or CerS6-deficient (**b,d**) naïve splenocytes. Panels e-h show histology of females that received adoptive transfer of wild type (**e,g**) or CerS6-deficient (**f,h**) naïve splenocytes. Histology is shown at 10x (**a**,**b,e**,**f**) and 20x (**c**,**d**,**g**,**h**) of the same section.

Upon euthanasia small and large intestines were collected to evaluate histology and to isolate cells from the lamina propria. Tissues were evaluated using a modified Dieleman scoring method that assessed multiple parameters as described in the Materials and Methods. In the small intestine of the wild type group, inflammatory infiltrate was evident in the muscularis and corresponded to mild or moderate inflammation. In the CerS6-deficient group the infiltrate was limited to the crypt base and was associated with mild or moderate inflammation severity. There was no increase in neutrophils and epithelial morphology was unremarkable within the small intestine of both groups. The number of lymphocytes recovered from the small intestine lamina propria was insufficient for flow cytometry analysis. Evidence of disease was more pronounced in the large intestine, where the total histological score was significantly higher in the wild type group (Fig. 3a). Inflammation was moderate to severe in the wild type group compared to generally minimal or mild in the CerS6-deficient group (Fig. 3b). In mice receiving wild type cells, extensive infiltrate reached the muscularis mucosae (with thickening of mucosa and abundant edema) or the submucosa. In contrast, in the CerS6-deficient group infiltrate tended to be limited to around crypt bases sometimes reaching the muscularis. Crypt inflammation was detected in half of the mice in the wild type group but not in the CerS6-deficient group. Crypt sloughed epithelial cells were significantly higher in the wild type compared to the CerS6-deficient group (Fig. 3c). Crypt sloughing is a pathological process that occurs when crypts become hyperplastic and epithelial cells that normally slough from the tip of the villi start sloughing into the lumen. This process may eventually lead to crypt abscesses. Epithelial morphology was also more affected following adoptive transfer of wild type rather than CerS6-deficient cells, although differences did not reach statistical significance (Fig. 3d). Adoptive transfer of wild type cells tended to result in moderate epithelial hyperplasia in the majority of mice (score 2 or 3), whereas mice receiving CerS6-deficient cells tended to have no or mild hyperplasia (score 0 or 1). Neutrophils were largely normal except for a slight increase in the colon of two mice that received wild type cells. Representative images of pathologies observed in wild type and CerS6-deficient mice are shown in Fig. 4.

**Figure 5:**
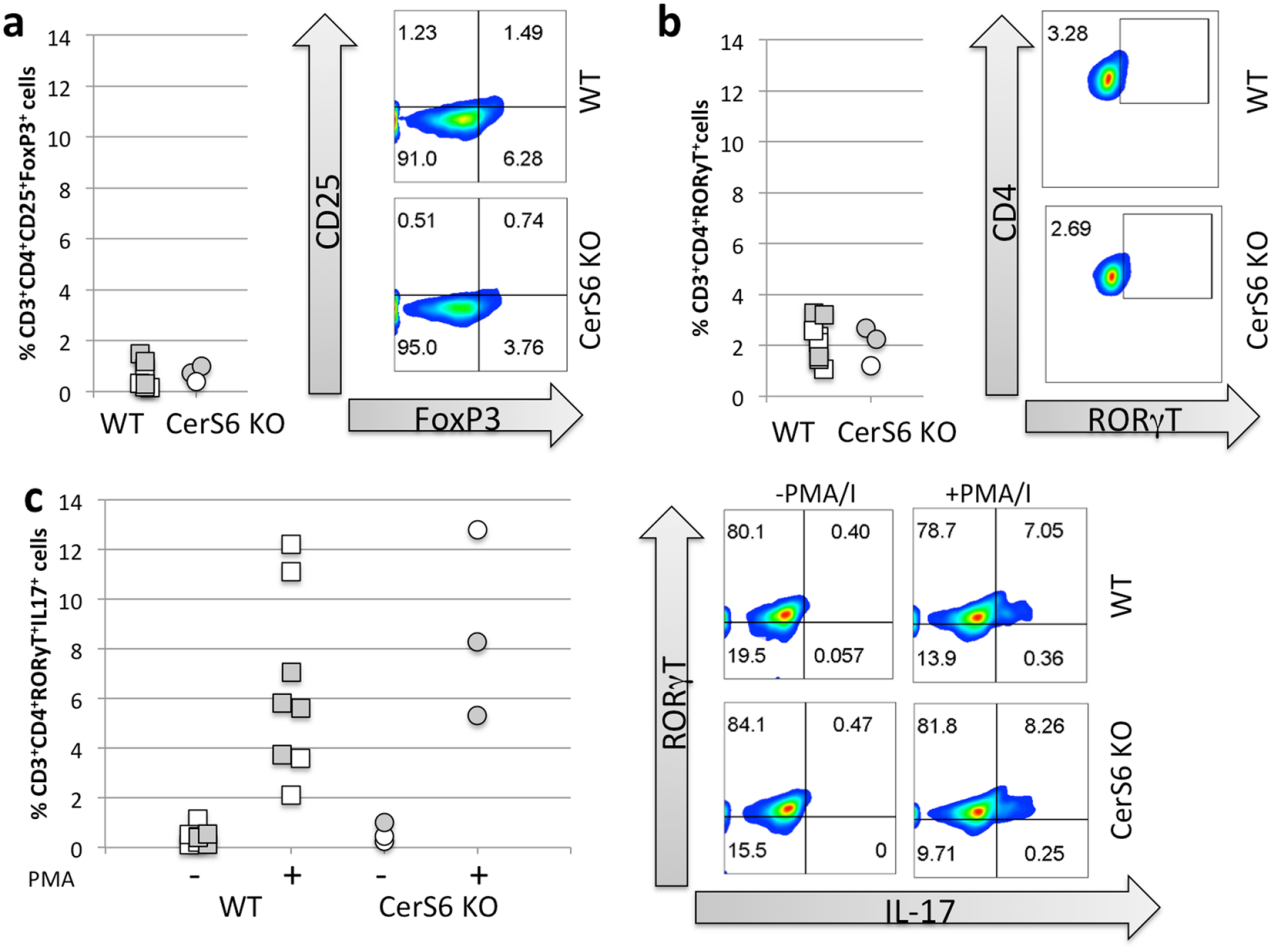
Analysis of colon lamina propria cells by flow cytometry. (**a**) Treg, (**b**) Th17, (**c**) IL-17 expression among Th17 cells that were unstimulated (−) or stimulated with PMA/ionomycin (+), n = 3–6. Males and females are indicated by white and grey symbols, respectively.

Next, we isolated cells from the gut lamina propria to determine potential differences in the proportion of immunosuppressive Treg or pro-inflammatory Th17 cells. The percentage of cells expressing the transcription factors FoxP3 and RORγT that are characteristic of immunosuppressive Treg and pro-inflammatory Th17 cells, respectively was comparable between the groups (Fig. 5a,b). No differences were detected in the ability of Th17 RORγT+ cells to express IL-17 (Fig. 5c). Interestingly, while sufficient cells for flow cytometry were recovered from all mice in the wild type group (8/8), we were able to recover cells from only 3 of 5 mice in the CerS6-deficient group. This did not appear to be a failure of the cells to populate recipients following adoptive transfer as neither spleen weight (p = 0.176) nor the number of splenocytes (p = 0.158) significantly differed between CerS6-deficient and wild type groups.

### Decreased C_16_-ceramide levels do not interfere with *in vitro* polarization into Treg or Th17 cells

**Figure 6:**
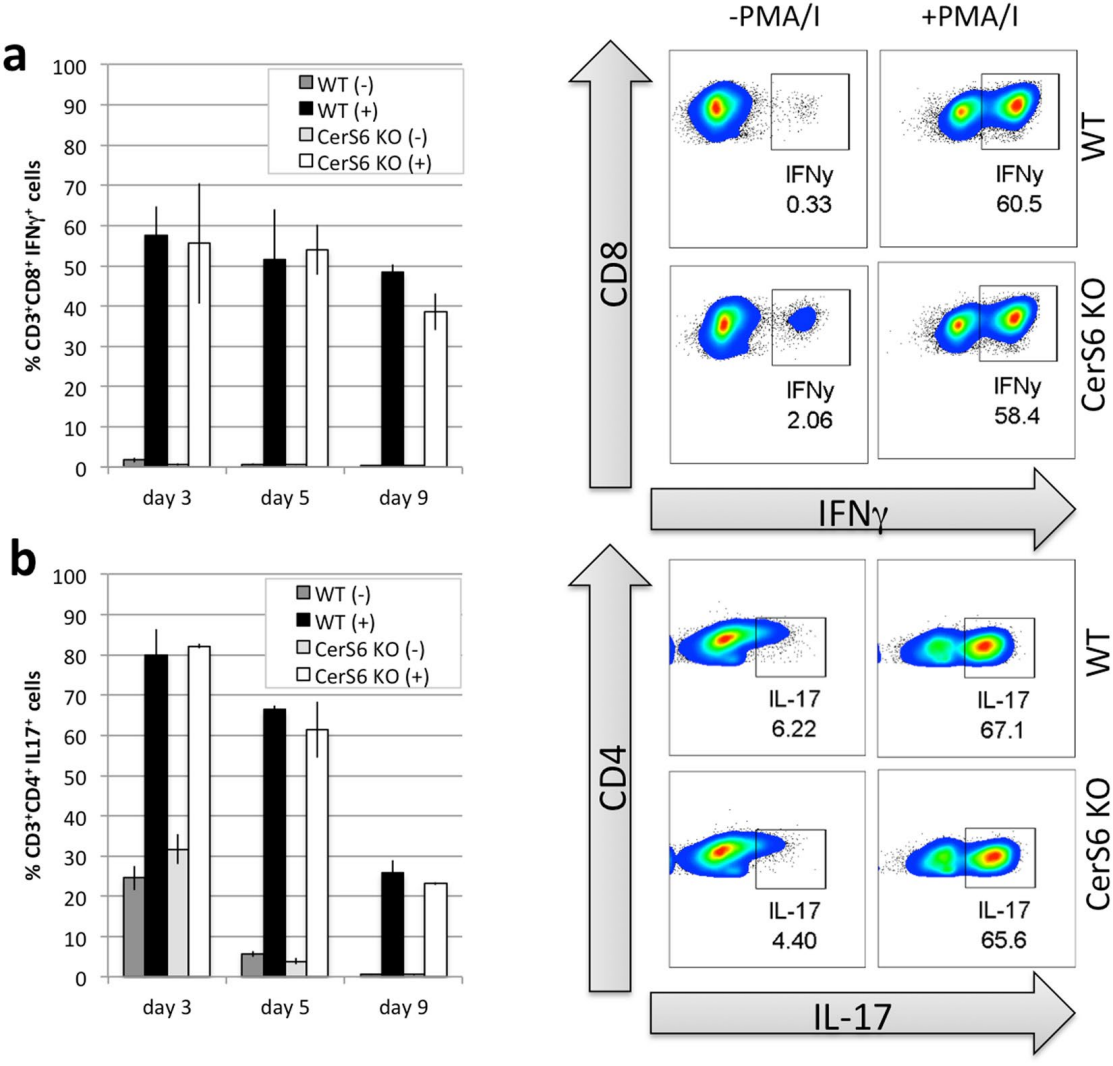
Polarization of wild type and CerS6-deficient splenocytes. Splenocytes were polarized towards (**a**) T1 or (**b**) T17 phenotype as described in the materials and methods. Flow cytometry was performed on days 3, 5, and 9 after polarization. Data represents the mean +/− SD, n = 2. Similar results were obtained in an independent experiment.

To confirm that CerS6-deficiency does not impact T cell subset development, we also polarized cells *in vitro*. Splenocytes were incubated under conditions known to drive differentiation towards T1, T17 and Treg subsets. Expression of the transcription factor Tbet, which regulates the development of naïve T lymphocytes was expressed under all polarizing conditions, while RORγT and FoxP3 were preferentially expressed under condition that favor the development of pro-inflammatory T17 cells and immunosuppressive Treg cells, respectively (Fig. S3). There were no significant differences among transcription factor expression between wild type and CerS6-deficient cells (Fig. S3). Both IFN- γ and IL-17 have been linked to the development of colitis. We therefore analyzed the ability of Th1 polarized CD3^+^CD8^+^ cells to express IFN-γ and Th17 CD3^+^CD4^+^ cells to express IL-17. Stimulation with PMA/ionomycin resulted in comparable induction of cytokine expression in both wild type and CerS6-deficient cells (Fig. 6). Taken together these results suggest that the decrease in C_16_-ceramide resulting from CerS6 deficiency does not impact the ability of cells to develop into T cell subsets or express cytokines such as IL-17 or IFN- γ.

**Figure 7:**
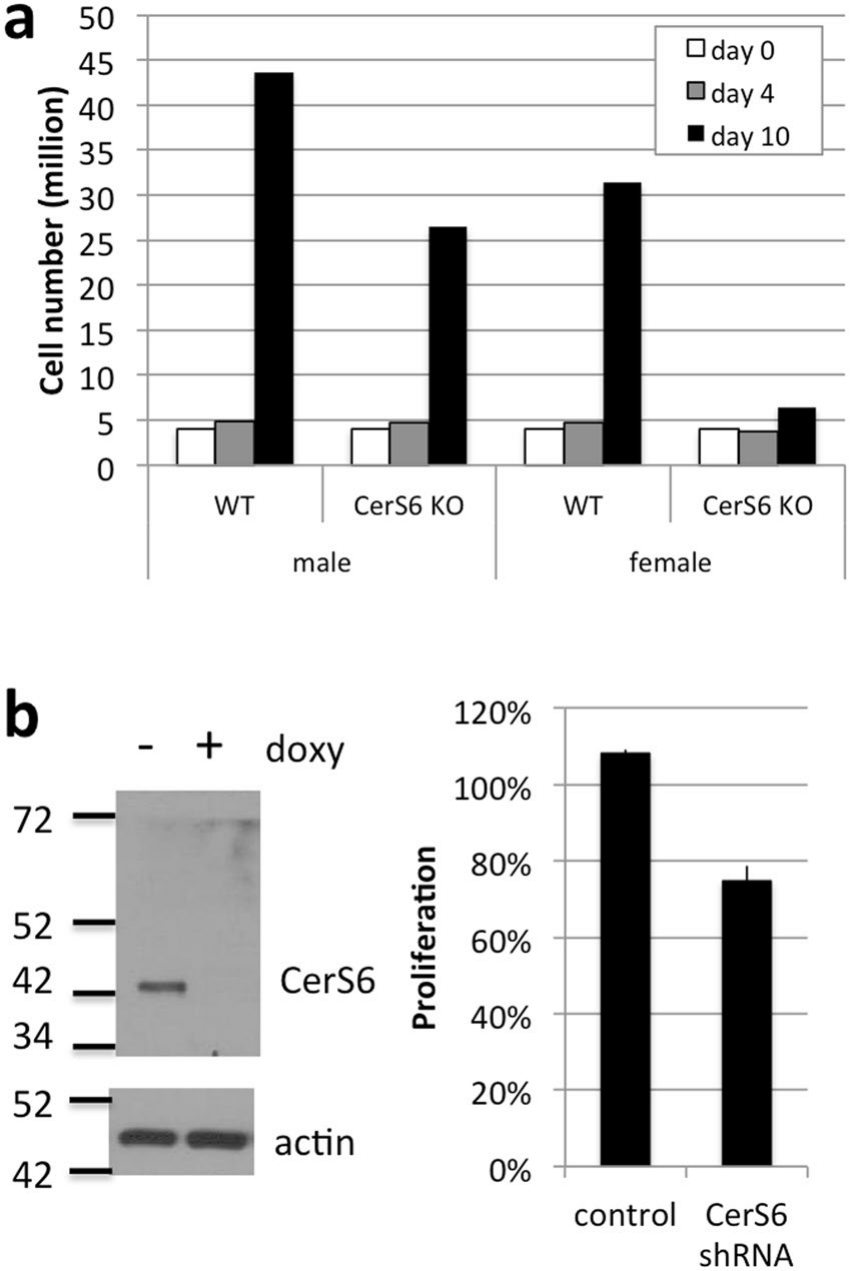
Effect of CerS6 ablation on proliferation. (**a**) Splenocytes from wild type and CerS6-deficient littermates were activated and clonal expansion assessed through cell counting. (**b**) SW480 cells −/+ shRNA against CerS6 were grown in the absence or presence of 10 μg/ml doxycycline for 4 days. Knockdown of CerS6 was verified by Western blot and proliferation assessed using the CellTiter-Blue assay. Values were normalized to cells grown in the absence of doxycycline. n = 2.

To evaluate whether CerS6 diminishes proliferative capacity, we activated splenocytes from wild type and CerS6-deficient mice (day 0) and counted cells on day 4 and 10. We found that CerS6-deficient cells expanded less by day 10 (Fig. 7a). To determine whether lack of CerS6 can diminishes proliferation in a non-T cell model, we evaluated SW480 colon cancer cells that express a doxycycline-inducible CerS6 shRNA^18^. As shown in Fig. 7b, treatment with doxycycline did not affect SW480 control cells but proliferation was reduced by approximately 25% in cells that expressed CerS6 shRNA.

## Discussion

Ceramide synthase 6, which preferentially generates C_16_-ceramide, has been implicated to play roles in various cellular responses^19^. CerS6-deficient mice were significantly smaller than their wild type littermates (Fig. 1a). Reduced body weight is likely due to an effect on fat metabolism as CerS6-deficient mice are protected from diet-induced obesity^13^. Transgenic mice that lack CerS5, which like CerS6 preferentially generates C_16_-ceramide, also maintain a lean phenotype on a high fat diet, although no significant difference in body weight was discernible on a low fat diet^12^. Our mice were fed a regular diet and therefore differences in body weight were subtle, becoming more apparent with age but clearly significant upon comparison of male littermates. Characterization of ceramide composition confirms that absence of CerS6 results in a specific decrease in C_16_-ceramide (Fig. 1, Table S1). In contrast to a recent study that reported a higher proportion of splenic CD8^+^ cells in CerS6-deficient mice, we did not observe any effect of a specific decrease in C_16_-ceramide on the proportions of splenic CD4^+^ or CD8^+^ T cells or on memory phenotype^20^. The underlying reason for this discrepancy is unclear.

Adoptive transfer of naïve CD4^+^ T cells resulted in significantly reduced colitis following adoptive transfer (Figs 2–4 *)*. These results are consistent with other recent studies in which CerS6-deficiency had a protective effect against inflammation. For example, transfer of CerS6-deficient T cells also led to reduced development of graft-vs-host disease and diminished macrophage infiltration and pro-inflammatory gene expression in the diet-induced obesity model^13,20^. In the graft-vs-host disease model, CerS6-deficiency interfered with allogenic T cell responses by reducing transduction of the TCR signal^20^. The mechanism by which CerS6-deficiency protects from inflammation in the diet-induced obesity model remains to be elucidated but was not directly mediated by macrophages as lineage-specific ablation of CerS6 failed to recapitulate the protective effect^13^. The pro-inflammatory lipid sphingosine-1-phosphate (S1P) has been associated with colitis and targeting S1P signaling has shown promising results in preclinical models^21^. However, since levels of S1P did not differ in splenocytes from wild type and CerS6-deficient mice, it is unlikely that altered S1P signaling contributed to the colitis phenotype observed in our study.

In patients with colitis, Th17 cells are increased while Treg are decreased, suggesting the proportion of these cells plays an important role in disease development^22^. The underlying reason for diminished development of colitis in our model does not appear to be a direct result of altered T cell subsets (i.e. more immunosuppressive Treg or less pro-inflammatory Th17 cells) or the ability to express the pro-inflammatory cytokine IL-17. Our data suggest that CerS6-deficiency may interfere with migration or proliferation *in vivo* rather than alter T cell subsets. In cancer cells, altered membrane fluidity in CerS6-deficient cells reduces cell migration and invasion of *in vitro* ^23,24^. Although mechanisms of motility between cancer cells and lymphocytes are different, it is possible that CerS6-deficient T cells have a reduced ability for homing and invasion of intestinal tissues. Our data suggest that CerS6-deficient splenocytes have a reduced capacity for clonal expansion. Reduced proliferation has been observed in CerS6-deficient T cells by Sofi *et al*.^20^. Expression of CerS6 shRNA also decreased the growth of SW480 cancer cells. However, loss of CerS6 does not universally decrease proliferation as targeting this enzyme results enhanced proliferation in melanoma cells^20,25^.

It is important to point out that CerS6-deficiency is not consistently protective against inflammation. In experimental autoimmune encephalomyelitis (EAE), an animal model of multiple sclerosis, CerS6-deficiency enhances inflammation through increased infiltration of neutrophils as CerS6 was not able to counteract CD11b mediated activation and CXCR2-mediated migration^11^. Thus the different consequences of CerS6 ablation in various models and cell types underscores the complexity of ceramide synthase signaling, which may depend on the overall sphingolipid environment in which ceramide synthase isoforms are modulated. Ceramide synthases differ not only in tissue distribution but also in subcellular localization and their ability to form homo- or heterodimers^4,19,26^. CerS2, -4, -5 and -6 activity can also be modulated through phosphorylation^27^. Thus regulation of CerS activity is multi-faceted and additional studies to understand the biology of ceramide synthases will be required before therapeutic strategies to modulate the activity of ceramide synthases can be rationally designed.

## Methods

### Animals

All animal experiments were performed with approval by the Institutional Animal Care and Use Committee at the Medical University of South Carolina to ensure that ethical regulatory and policy mandates governing the use of animals in research are met (Animal Welfare Assurance #A3428-01). All methods were performed in accordance with the relevant guidelines and regulations. CerS6-deficient mice (C57BL6) were generated by the Texas Institute of Genomic Medicine and obtained from Dr. Besim Ogretmen. Colonies were maintained through heterozygous breeding. Genomic tail DNA was used to genotype offspring by PCR. Primers were as follows: 5′TTCGGTTAAGAATGGCCTTG3′; 5′CACACCCATATGGAACTCTTACA-3′; and 5′-CCAATAAACCCTCTTGCAGTTGC-3′. Expected PCR products are 460 bp for wild type, 295 bp for CerS6 knockout, or both for heterozygous animals. RAG1-deficient mice (B6.129S7-Rag1tm1Mom/J) were purchased from Jackson Laboratories.

### Ceramide analysis

Ceramide species (C_14_, C_16_, C_18_, C_18:1_, C_20_, C_20:1_, C_20:4_, C_22_, C_22:1_, C_24_, C_24:1_, C_26_ and C_26:1_ were quantified by LC/MS as previously described^18^.

### Cell isolation, polarization, and adoptive transfer

Four million wild-type or CerS6-deficient splenocytes/well in a 24-well plate were polarized in 2 ml RPMI with 10% FBS (Hyclone) and 1 μg/ml anti-mouse CD3 as follows: For T1 polarization, 6ng/ml mIL-12, 20 μg/ml anti–mouse anti–mouse IL-4 (anti-mIL-4, clone 11B11), and 200 IU/ml hIL-2 were added. For T17 polarization 100 ng/ml recombinant human (rh) IL-6 (NIH repository), 100 ng/ml rhIL-21 (Shenandoah), 30 ng/ml rhTGF-β1 (Biolegend), 10 ng/ml rhIL-1β (NIH Repository), 10 μg/ml each of anti–mIFN-γ (clone XMG1.2), anti–mIL-4 (clone 11B11), and anti–mIL-2 (clone JES6-1A12) (all BioXCell) were added. For Treg we added 2 μM rapamycin, 60 ng/ml rhTGF-β1 (Biolegend), and 200 IU/ml IL-2. On day 3, 1 ml of the supernatant was removed without disturbing the cells and 20 ng/ml IL-23 and 50 IU/ml IL-2 or 200 IU/ml IL2 were added to Th17 and Treg cultures, respectively. On day 4 cells were counted and placed into fresh RPMI with 10% FBS and 100 IU/ml IL2. Flow cytometry was performed 3, 5- or 9 days after polarization.

For adoptive transfer, splenocytes from wild type or CerS6 deficient mice were pooled and naïve CD4^+^ mouse splenocytes obtained through deletion of non-target cells (Naïve CD4^+^ T cells isolation kit, Miltenyi Biotec 130-104-453) followed by further selection for CD45RB^HI^ cells (Biolegend) as previously described^28^. Prior to adoptive transfer, flow cytometry was used to verify that populations contained at least 95% of desired cells. 200,000 cells/animal were injected into RAG-1-deficient recipients via retro-orbital route. Data in this manuscript were from 2 independent experiments performed with a total of 10 wild type and 8 CerS6-deficient cell recipients.

Isolation of lamina propria cells was performed as previously described^29^. Briefly, after small and large intestines were cleaned, a small section of tissue was collected for histology and the remainder digested (2 mg/ml collagenase D, 0.1 mg/ml DNAse I and 0.075 U/ml dispase), and washed. Lymphcytes were enriched by Percoll centrifugation and cells at the interface collected for subsequent analysis.

### Flow cytometry

Cells were incubated with or without PMA/ionomycin in Golgi-Stop for 4 hours and then surface stained with fluorochrome-conjugated antibodies against CD3, CD4, and CD25 to allow for gating of specific populations. For subsequent intracellular staining, surface stained cells were washed, fixed in 4% paraformaldehyde for 20 minutes, and permeabilized for 30 minutes prior to overnight incubation with antibody (FoxP3, RORγT, IL-17, IFN-γ). After washing, signals were acquired using the BD LSRFortessa cell analyzer (BD Biosciences) and analyzed with the FlowJo software (Tree Star Inc.). Treg were defined as CD3^+^CD4^+^CD25^+^FoxP3^+^ and Th17 cells as CD3^+^CD4^+^ RORγT^+^.

### Histology analysis

Intestinal tissues were removed and fixed in formalin. Sections were placed in cassettes and cut longitudinally for histological analysis. Tissues were scored as follows: lymphoid follicles (0, none; 1, one; 2, two; 3, three; 4, greater than 3); infiltration of inflammation (0, none; 1, around crypt bases; 2, infiltrate to muscularis; 3, extensive infiltrate reaching muscularis mucosae with thickening of the mucosa with abundant edema); epithelium (0, normal morphology; 1, loss of goblet cells; 2, loss of goblet cells in a large area; 3, loss of crypts; 4, loss of crypts in a large area/polypoid formation); depth of ulcer (0, none; 1, mucocal involvement; 2, mucosal, submucosal; 3, penetration of muscularis propria; 4, full thickness); extent of ulcer (0, none; 1, punctate; 2, minimal; 3, moderate; 4, widespread); crypt sloughed cells (0, none; 1, 1 cell; 2, 2–5 cells; 3, many); inflammation severity (0, none; 1, minimal; 2, mild; 3, moderate; 4, marked – severe)^30–32^.

### Statistical Analysis

Paired (Fig. 1a) or two-sample t-tests (Figs 1b,d and 3) were used to compare means between wild type and CerS6-deficient groups. Statistical significance was defined by a two-sided alpha level of 0.05. In Fig. 2, relative body weight (weight divided by baseline weight) was modeled over time using linear regression, estimated using generalized estimating equations to account for repeated measures over time. Based on exploratory data analysis, there appeared no need to transform the outcome measure. The fitted model included treatment effects, a linear term for time, and also a quadratic time term to account for the non-linear pattern over time, and interactions between time main effect and treatment groups. Linear combinations of model coefficients were used to evaluate differences between groups at individual time points, using Wald tests and 95% confidence intervals.

## Electronic supplementary material

Supplementary info
