# Supplementary material for: Adoptive Transfer of Ceramide Synthase 6 Deficient Splenocytes Reduces the Development of Colitis
Source: Sci Rep. 2017 Nov 14;7:15552. doi: 10.1038/s41598-017-15791-x (PMC5686186; doi:10.1038/s41598-017-15791-x)
Supplement: Supplementary file 1 — Supplementary info [file 41598_2017_15791_MOESM1_ESM.pdf]

Title:
Adoptive Transfer of Ceramide Synthase 6 Deficient Splenocytes Reduces the Development of Colitis

Authors:
Matthew J. Scheffel[1], Kristi Helke[2], Ping Lu[1], Jake Bowers[1], Besim Ogretmen[3], Elizabeth Garrett-Mayer[4], Chrystal M. Paulos[1], Christina Voelkel-Johnson[1]*

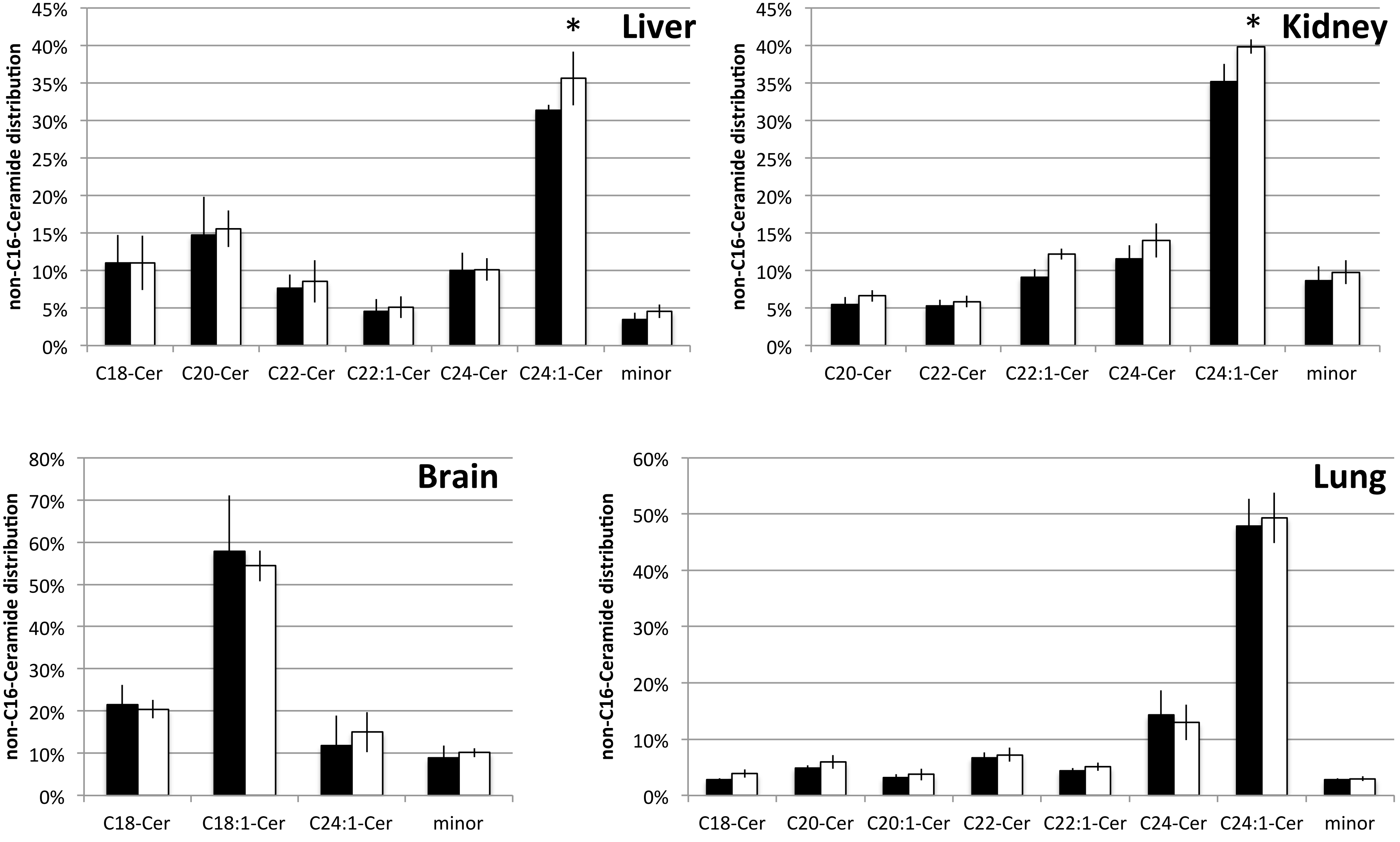

Figure S1. Ceramide distribution. The percentage of non-C_{16}-ceramides in various tissues is shown. Ceramide species representing less than 2% of the total were grouped and are shown as “minor”. A two-sample t test was used to calculate statistical significance. n=4-5. The C_{16}-ceramides of this set of data is shown in Fig. 1b of the manuscript. Closed bars are wild type and open bard CerS6 KO mice.

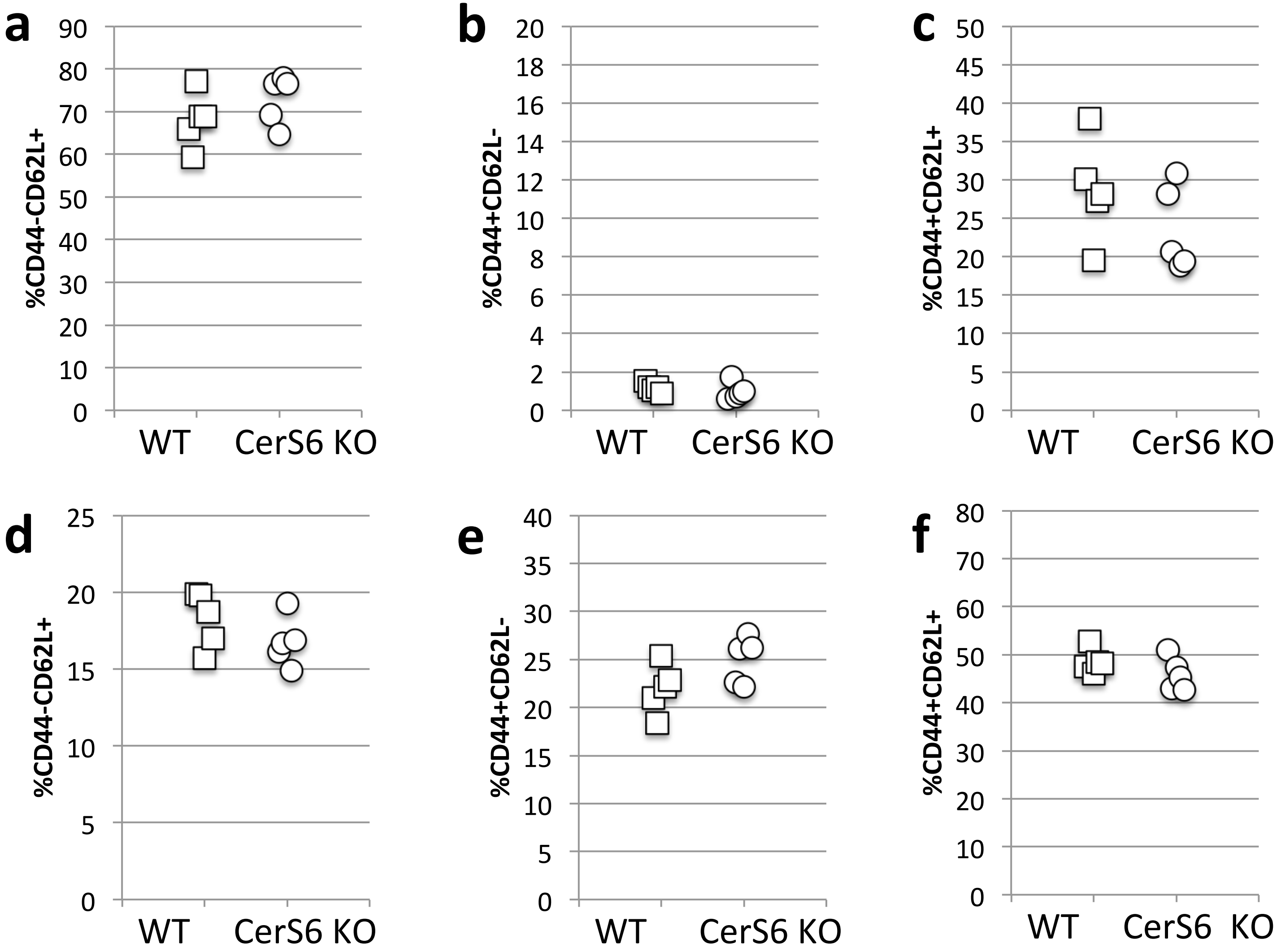

<u>Figure S2. Splenocytes memory phenotyp</u>e. Naïve splenocytes were isolated from wild type and CerS6-deficient littermates, processed, counted and surface stained with antibodies against CD3, CD4, CD8, CD44 and CD62L. Populations were gated on CD3/CD4 (a-c) or CD3/CD8 (d-f). No differences were found in between wild type and CerS6 deficient naïve T cells (CD44-CD62L+) (a, d), effector memory T cells CD44+CD62L- (b, e) and or double positive CD44+CD62L+ (c, f). N=5. Similar results were observed in an independent experiment.

a

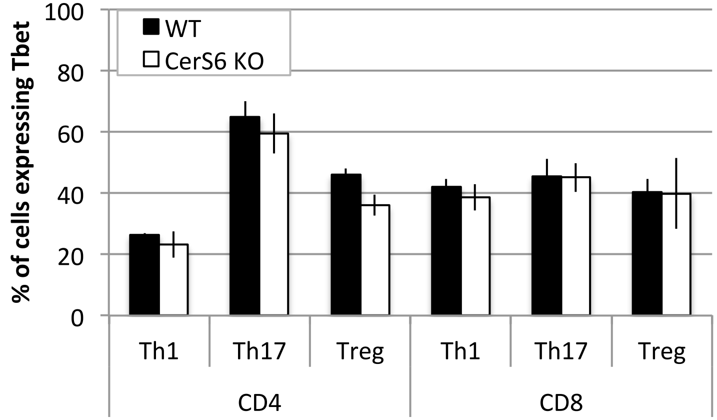

b

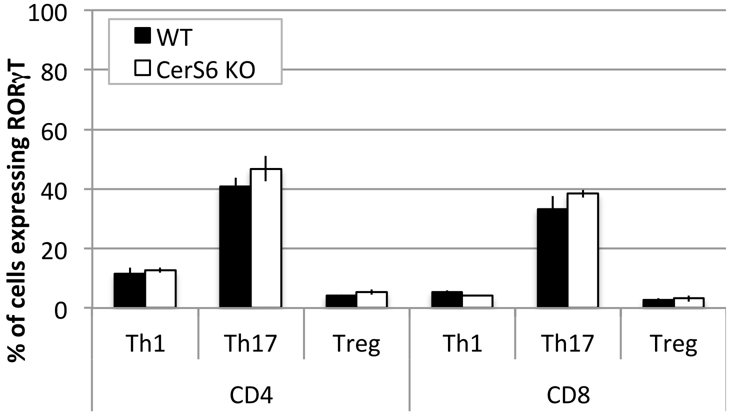

c

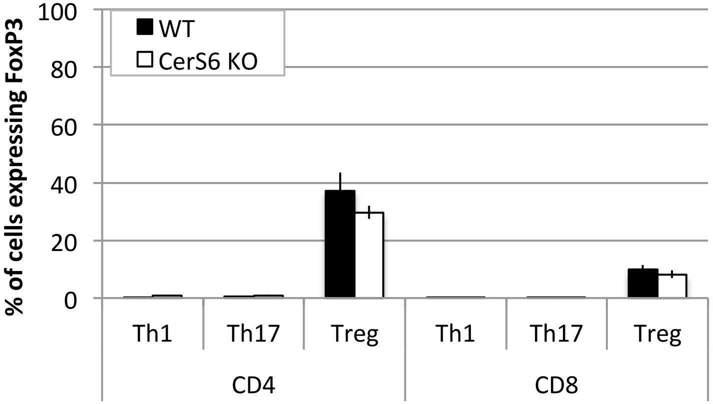

<u>Figure S3. Transcription factor expression following polarization</u>. Naïve splenocytes were isolated from 2 wild type and 2 CerS6-deficient littermates, processed, counted and 4 million cells of each culture polarized as described in the materials and methods. On day 4, cells were surface stained with antibodies against CD3, CD4, CD8, permeabilized and intracellularly stained for the transcription factors Tbet (a), RORγT (b) or FoxP3 (c). Expression of RORγT and FoxP3 was preferential in cells that had been polarized towards the T17 and Treg lineage, respectively. No significant differences were observed between wild type and CerS6-deficient cells. Similar results were observed in an independent repeat of this experiment.

	C14	C16	C18	C18:1	C20	C20:1	C20:4	C22	C22:1	C24	C24:1	C26	C26:1
**Liver (WT)**	15.9 ± 4.0	230.9 ± 61.1	137.2 ± 46.3	12.9 ± 4.6	183.8 ± 63.1	11.4 ± 2.0	13.4 ± 9.7	95.0 ± 23.4	57.0 ± 20.3	125.3 ± 28.7	391.1 ± 9.6	3.2 ± 0.7	2.4 ± 0.3
**Liver (CerS6 KO)**	8.5± 3.5*	111.2 ± 20.1**	132.5 ± 47.6	14.4 ± 4.5	205.4 ± 35.0	14.3 ± 2.2	18.6 ± 8.0	112.5 ± 41.9	69.8 ± 19.4	134.3 ± 20.3	461.9 ± 53.0*	4.8 ± 2.5	3.4 ± 1.4
**Kidney (WT)**	10.3± 0.7***	317.5± 55.6***	24.1 ± 6.8	24.0 ± 5.2	70.6 ± 12.8	37.2 ± 8.7	7.8 ± 3.4	67.4 ± 11.2	117.3 ± 14.3	148.9 ± 22.5	452.1 ± 30.2	4.5 ± 1.2	3.3 ± 0.3
**Kidney (CerS6 KO)**	5.2 ± 0.9	156.0 ± 18.8	33.2 ± 8.3	29.8 ± 5.4	88.5 ± 10.4	46.2 ± 10.6	6.5 ± 2.9	78.4 ± 10.7	162.6 ± 9.7**	186.6 ± 30.3	531.4 ± 13.0**	5.5 ± 2.7	3.9 ± 0.4
**Brain (WT)**	3.5 ± 1.7	37.1 ± 13.7	354.5 ± 90.6	1122.9 ± 259	29.6 ± 10.3	38.1 ± 4.5	6.6 ± 3.1	22.7 ± 11.3	16.0 ± 4.1	26.0 ± 20.6	297.6 ± 140	1.3 ± 0.7	3.3 ± 2.7
**Brain (CerS6 KO)**	4.3 ± 0.2	29.7 ± 6.0	395.2 ± 42.1	1053.0 ± 71.6	34.9 ± 2.3	48.0 ± 2.1	8.7 ± 4.4	28.2 ± 5.6	16.4 ± 3.6	22.8 ± 4.7	291.2 ± 92.5	1.0 ± 0.2	2.8 ± 0.8
**Lung (WT)**	1.9 ± 0.4	55.1 ± 7.4	11.9 ± 1.2	5.6 ± 0.9	20.7 ± 1.9	13.3 ± 2.6	0.3 ± 0.0	28.1 ± 3.9	18.4 ± 2.0	60.1 ± 18.5	201.1 ± 20.5	1.7 ± 0.5	2.3 ± 0.5
**Lung (CerS6 KO)**	1.2 ± 0.2*	42.5 ± 4.3*	18.6 ± 3.6	8.4 ± 2.2	28.7 ± 6.1	18.0 ± 5.2	34.9 ± 0.2	28.1 ± 5.9	24.6 ± 3.6	62.5 ± 15.2	237.6 ± 21.3	1.9 ± 0.9	2.4 ± 0.5
**Splenocytes (WT)**	0.6 ± 0.3	14.7 ± 1.7	5.8 ± 0.1	0.7 ± 0.1	4.1 ± 0.4	0.4 ± 0.0	0.1 ± 0.1	5.7 ± 1.1	1.2 ± 0.5	8.6 ± 3.7	23.7 ± 6.0	0.8 ± 0.0	0.4 ± 0.2
**Splenocytes (CerS6 KO)**	0.5 ± 0.2	8.6 ± 1.2	7.6 ± 1.7	0.8 ± 0.2	4.3 ± 1.4	0.3 ± 0.0	BDL	8.1 ± 2.3	1.4 ± 0.0	12.5 ± 2.6	26.8 ± 0.6	0.7 ± 0.4	0.3 ± 0.1

Supplementary Table 1. Distribution of ceramide species in tissues and splenocytes. Tissue or splenocytes from 4-5 mice were analyzed by LC-MS. For tissue data is expressed as pmol ceramide/mg protein (*1000). For splenocytes, data is expressed as pmol ceramide /5 million cells. BDL=below detectable levels. *p<0.05, **p<0.005, ***p<0.0005.
